# Supplementary material for: Enhanced Aerosol Containment Performance of a Negative Pressure Hood with an Aerodynamic Cap Design: Multi-Method Validation Using CFD, PAO Particles, and Microbial Testing
Source: Bioengineering (Basel). 2025 Jun 9;12(6):624. doi: 10.3390/bioengineering12060624 (PMC12189663; doi:10.3390/bioengineering12060624)
Supplement: Supplementary file 1 [file bioengineering-12-00624-s001.zip › bioengineering-3665103-supplementary.pdf]

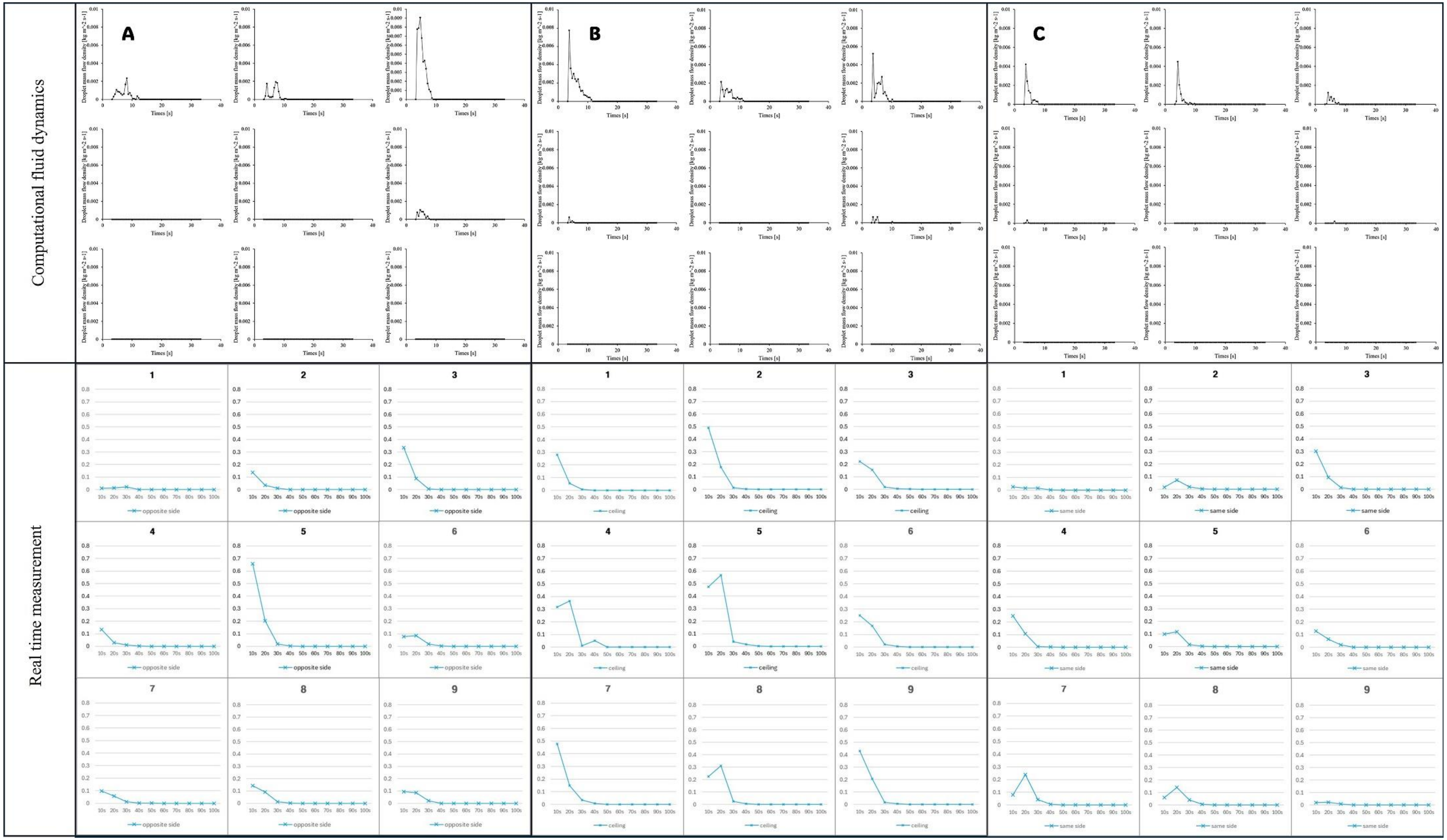

Supplementary Figure 1[1]. CFD simulation of droplet leakage and real-time measurement of droplet leakage. (A) Coughing directed

toward the opposite side, (B) coughing directed toward the ceiling, and (C) coughing directed toward the same side as the suction hole. The overall leakage patterns observed in the CFD analysis are similar to those obtained through real-time measurements.

Supplementary Table [1]1. Average and Standard deviation of droplet leakage over time (without cap)

Time	Brething condiotin						Coughing condiotin					
	Ceiling		Same side		Opposite side		Ceiling		Same side		Opposite side	
(sec)	Mean	SD	Mean	SD	Mean	SD	Mean	SD	Mean	SD	Mean	SD
10	0.0536	0.0786	0.0096	0.0350	0.0111	0.0245	0.3067	0.2822	0.1791	0.2668	0.1172	0.2208
20	0.0288	0.0803	0.0001	0.0004	0.0084	0.0193	0.2093	0.3634	0.0694	0.0810	0.1045	0.1277
30	0.0074	0.0253	0.0000	0.0001	0.0081	0.0289	0.0188	0.0170	0.0135	0.0137	0.0222	0.0250
40	0.0007	0.0030	0.0000	0.0001	0.0028	0.0057	0.0052	0.0334	0.0015	0.0019	0.0026	0.0044
50	0.0000	0.0000	0.0000	0.0000	0.0031	0.0144	0.0009	0.0020	0.0002	0.0003	0.0000	0.0000
60	0.0000	0.0000	0.0006	0.0033	0.0037	0.0137	0.0002	0.0004	0.0000	0.0000	0.0000	0.0000
70	0.0000	0.0000	0.0000	0.0002	0.0015	0.0070	0.0000	0.0001	0.0000	0.0000	0.0000	0.0000
80	0.0000	0.0000	0.0000	0.0001	0.0031	0.0167	0.0000	0.0000	0.0000	0.0000	0.0000	0.0000
90	0.0000	0.0000	0.0000	0.0000	0.0053	0.0317	0.0000	0.0000	0.0000	0.0000	0.0000	0.0000
100.	0.0000	0.0000	0.0000	0.0000	0.0000	0.0000	0.0000	0.0000	0.0000	0.0000	0.0000	0.0000

1. Hong, J.Y., et al., *Enhancing Aerosol Mitigation in Medical Procedures: A CFD-Informed Respiratory Barrier Enclosure.* Bioengineering, 2024. **11**(11): p. 1104.
